# How Ohio's proposed abortion bans would impact travel distance to access abortion care

**DOI:** 10.1363/psrh.12191

**Published:** 2022-04-20

**Authors:** Payal Chakraborty, Stef Murawsky, Mikaela H. Smith, Michelle L. McGowan, Alison H. Norris, Danielle Bessett

**Affiliations:** ^1^ Division of Epidemiology, College of Public Health The Ohio State University Columbus Ohio USA; ^2^ Department of Sociology, College of Arts and Sciences University of Cincinnati Cincinnati Ohio USA; ^3^ Ethics Center Cincinnati Children's Hospital Medical Center Cincinnati Ohio USA; ^4^ Department of Pediatrics, College of Medicine University of Cincinnati Cincinnati Ohio USA; ^5^ Department of Women's, Gender & Sexuality Studies, College of Arts and Sciences University of Cincinnati Cincinnati Ohio USA; ^6^ Division of Infectious Diseases, College of Medicine The Ohio State University Columbus Ohio USA

## Abstract

**Context:**

Since March 2021, the Ohio legislature has been actively considering laws that would ban abortion if the United States Supreme Court overturns the *Roe v. Wade* decision that legalized abortion nationally in 1973.

**Methods:**

We used a national database of publicly advertised abortion facilities to calculate driving distances for Ohioans before and after the activation of proposed abortion bans. Using a legal analysis of abortion laws following the overturn of *Roe*, we determined which states surrounding Ohio would continue providing abortion care. We calculated distances from each Ohio county centroid to the nearest open abortion facility in three scenarios: (1) as of February 2022, (2) the best‐case post‐*Roe* scenario (two of the five surrounding states continue to offer abortion care), and (3) worst‐case post‐*Roe* scenario (no surrounding states continue to offer abortion care). We calculated population‐weighted distances using county‐level data about women aged 15–44 years from the 2019 American Community Survey.

**Results:**

In February 2022, all Ohio county centroids were at most 99 miles from an abortion facility (median = 50 miles). The best‐case post‐*Roe* scenario shows 62 of Ohio's 88 counties to be 115–279 miles away from the nearest facility (median = 146). The worst‐case shows 85 counties to be 191–339 miles away from the nearest facility (median = 264). The current average population‐weighted driving distance from county centroid to the nearest facility is 26 miles; the post‐*Roe* scenarios would increase this to 157 miles (best‐case) or 269 miles (worst‐case).

**Conclusions:**

Ohio's proposed abortion bans would substantially increase travel distances to abortion care, impacting over 2.2 million reproductive‐aged Ohioans.

## INTRODUCTION

The Supreme Court of the United States (US) first recognized the constitutional right to abortion in the 1973 case *Roe v. Wade* (commonly referred to as *Roe*). This landmark decision prohibited states from banning abortion prior the third trimester of pregnancy; after this point, the state could regulate or prohibit abortions except where care was needed “for the preservation of the life or health of the mother.”^1^ In 1992, *Planned Parenthood v. Casey* asked the US Supreme Court to decide the constitutionality of state restrictions on abortion throughout pregnancy, including in the first trimester.^1^ The US Supreme Court ruled to protect the essence of *Roe*, that is, uphold the individual right to abortion, but to allow a number of state restrictions.^1^ Specifically, states could regulate abortion before viability (approximately 24 weeks gestation) so long as the regulation in question did not place an “undue burden…in the path of a woman seeking an abortion.”^1^, ^2^

Once the US Supreme Court established the right to abortion, as well as how states could regulate the practice, many states began passing laws to restrict the circumstances under which people could obtain abortion care. These state‐level restrictions directly affected abortion seekers (e.g., restricting use of public insurance to pay for abortions, enacting mandatory waiting periods) and specifically targeted abortion providers (e.g., requiring clinics to establish medically unnecessary agreements with hospitals to transfer patients or to comply with medically unnecessary building requirements). The current accessibility of abortion in the United States thus varies by state and is characterized by a patchwork of service availability resulting in vast abortion deserts, or large geographic areas where abortion is unavailable.^3^

The implementation of existing abortion restrictions has coincided with facility closures, increased wait times, and differences in care offerings,^4^, ^5^, ^6^, ^7^, ^8^, ^9^ creating barriers to care, including long travel distances for abortion seekers. Current estimates of interstate travel suggest between 6% and 7% of patients already cross state lines to receive abortion care.^10^, ^11^, ^12^ The consequences of longer travel distance for abortion care are well established, including increased costs,^5^, ^13^ delays to care,^14^, ^15^ inability to receive one's preferred method of care,^16^ and increased stress.^15^ In this way, increased travel for abortion care creates unnecessary barriers to health care.^10^, ^17^ Such barriers are sometimes insurmountable and lead to undesired continued pregnancies. Additionally, they are inequitably experienced,^10^, ^18^, ^19^ adding to the increased health inequities experienced generally by people with fewer financial resources and people of color.^20^

In addition to state laws determining how abortion care is provided, states have passed laws banning abortion, including pre‐*Roe*, pre‐viability, pregnancy duration, method, reason, and trigger bans.^21^ Pre‐*Roe* bans are abortion laws that were established prior to 1972 and became unenforceable after the ruling in *Roe*, yet remain on the books. Pre‐viability bans attempt to prohibit abortion before a fetus is considered viable. Pregnancy duration bans, which seek to bar abortion after a certain point in a pregnancy (e.g., 6‐week, 12‐week, or 20‐week bans) or after the detection of specific embryonic or fetal development markers (e.g., “heartbeat” bans), are common. Method bans proscribe certain abortion procedures, such as dilation and extraction (D&X) or dilation and evacuation (D&E). Reason bans disallow abortions sought for specific reasons, such as the presence of genetic anomalies, or because of presumptive sex or race. In addition, a number of states have passed “trigger bans,” that is complete or severe bans on abortion that are contingent on and would be “triggered” by the US Supreme Court overturning or weakening *Roe*. As of February 2022, 12 states had passed laws that would prohibit abortion throughout pregnancy if *Roe* falls: Arkansas, Idaho, Kentucky, Louisiana, Mississippi, Missouri, North Dakota, Oklahoma, South Dakota, Tennessee, Texas, and Utah.^21^ Trigger laws and other types of outright bans would be enforceable only if *Roe* is overturned; providers in states with such laws would no longer be able to offer abortion care. Residents of these states seeking an abortion would need to travel across state lines for care within the formal health system.

While many ban types are currently considered unconstitutional, the US Supreme Court will soon issue a decision in *Dobbs v. Jackson Women's Health Organization* that could significantly change the legal abortion landscape. Indeed, some legal analysts expect that the US Supreme Court will either overrule or severely curtail *Roe*.^22^, ^23^ The consequences of such a ruling could allow the reconsideration and enactment of state abortion bans that are currently considered unconstitutional and could have profound impacts on abortion access, amplifying challenges experienced by abortion seekers and increasing the negative health, financial, and social outcomes associated with being denied a wanted abortion.^24^, ^25^, ^26^, ^27^, ^28^, ^29^

Ohio is considering multiple options for severely restricting abortion care should *Roe v. Wade* be overturned. The Ohio State Senate introduced trigger ban legislation in March 2021, entitled Senate Bill (SB) 123 or the “Human Life Protection Act,” and the Ohio House, the other chamber of the Ohio General Assembly, put forth a parallel bill in March 2022, entitled House Bill (HB) 598. These laws are functionally identical, just introduced separately by the House and the Senate. In addition, the Ohio General Assembly introduced HB 480 in November 2021, entitled “Authorize Private Right of Action for Abortion or Aiding Abortion,” which would allow private citizens to sue individuals inducing or aiding in an induced abortion. These bills are being actively considered in the state legislature, could pass at any time, and are likely to be signed into effect by the governor should they pass.

In this manuscript, we document how far patients from Ohio, an abortion‐hostile state,^30^, ^31^ would need to travel if legislation banning abortion or creating a private right of civil action in Ohio were to pass and *Roe* were overturned or weakened. We demonstrate the anticipated impacts of SB 123/HB 598 and/or HB 480 by calculating and comparing the current distance Ohioans must go to reach the nearest abortion facility, by county, and the distance to the nearest out‐of‐state facility likely to offer services in several post‐*Roe* scenarios, where SB 123/HB 598 and/or HB 480 are in effect. We also examine how expected changes in travel distances and their associated costs vary by region of the state and subpopulation. Finally, we consider the demographic characteristics of five counties (Lucas, Hamilton, Montgomery, Franklin, and Cuyahoga) to illuminate which sub‐populations would be most impacted by an Ohio abortion ban.

## METHODS

### Setting

Ohio is a state located in the Midwestern region of the US, bordered by Indiana, Kentucky, West Virginia, Pennsylvania, Michigan, and Lake Erie. It has the seventh highest population among US states, at about 11.8 million people.^32^ Ohio has a highly restrictive abortion policy climate. As of February 2022, some of Ohio's abortion restrictions included an in‐person facility visit followed by a 24‐h waiting period, a 22‐week gestational limit, limits on use of public health insurance for abortion, a reason ban for fetal diagnosis or indication of Down syndrome, parental consent and judicial bypass requirements, and written transfer agreement requirements for abortion facilities.^33^, ^34^ Ohio lawmakers also passed a six‐week gestation ban, which is currently blocked by the federal court, but could go into effect in a post‐*Roe* scenario. SB 123/HB 598 and/or HB 480 would threaten abortion access in Ohio further. Most of Ohio's neighboring states have multiple abortion restrictions and generally hostile abortion policy climates.^35^

### Analytic approach

To calculate one‐way driving distance before and after enactment of SB 123/HB 598 and/or HB 480, we used Advancing New Standards in Reproductive Health's (ANSIRH's) Abortion Facility Database.^36^ This database includes all publicly identifiable US abortion facilities that currently offer medication and/or instrumentation abortion care. Our analysis examines three scenarios: one scenario (A) represents the travel distance from county centroids to the nearest abortion facilities in February 2022, and two scenarios (B and C) depict the best‐ and worst‐case scenarios for the projected travel distances from county centroids to the nearest out‐of‐state facilities if abortion bans in Ohio were to go into effect. We used ArcGIS programs (ESRI, Redlands, CA) to calculate current driving distance (using road maps as of January 2022) from each Ohio county centroid to the closest abortion facility (in Ohio or out‐of‐state), as well as the distance to the nearest open facility in the two post‐*Roe* scenarios where any of the above abortion bans are in effect. For Scenario A (February 2022 driving distances) we excluded Ohio facilities that dispense the mifepristone/misoprostol regimen of medication abortion only via telemedicine, as patients still need to travel to another abortion facility for pre‐abortion counseling and informed consent.^37^

In Scenarios B and C, in which *Roe v. Wade* is overturned/substantially curtailed and an Ohio abortion ban is in effect, we considered the landscape of abortion access in Ohio's neighboring states. Specifically, we assessed which of Ohio's neighboring states would also ban abortion if *Roe* were overturned. We did so using the Center for Reproductive Rights' analysis of state‐level projected post‐*Roe* political landscapes and hostility,^21^ following Myers and colleagues' methodology,^38^ which also used this policy analysis to consider driving distance to abortion facilities. Many of Ohio's bordering states would be considered hostile to abortion in a post‐*Roe* scenario: Indiana lawmakers are expected to prohibit abortion, Kentucky has already passed a trigger ban, Michigan has a pre‐*Roe* ban, and West Virginia has a state constitutional amendment indicating no right to abortion. Abortion would likely remain accessible in some states adjacent to Ohio's bordering states. Specifically, Illinois and Maryland have abortion protections in place, and Virginia, although it does not have abortion protections, is unlikely to immediately pass a trigger law or a post‐*Roe* ban. It remains uncertain whether two of Ohio's neighboring states, Pennsylvania and Michigan, would implement abortion bans in a post‐*Roe* scenario. In Pennsylvania, abortion is not legally protected. Pennsylvania legislators are expected to pass laws banning abortion, but the current governor is unlikely to sign such laws; thus, abortion access in post‐*Roe* Pennsylvania would be dependent on a governor veto. In Michigan, it is unclear if the current governor will be successful at stopping enforcement of its pre‐*Roe* ban, the legal status of which remains uncertain in a post‐*Roe* scenario. Thus, in this analysis we consider a “best‐case” scenario (Scenario B), where abortion remains accessible in Pennsylvania and Michigan, and a “worst‐case” scenario (Scenario C), where abortion is banned in both Pennsylvania and Michigan. In this analysis, we calculated driving distances assuming patients travel to the geographically closest facility. In Scenario B, this would be to facilities in Illinois, Michigan, Virginia, and Pennsylvania; in Scenario C, this would only be to facilities in Illinois, Virginia, and Maryland.

**FIGURE 1:**
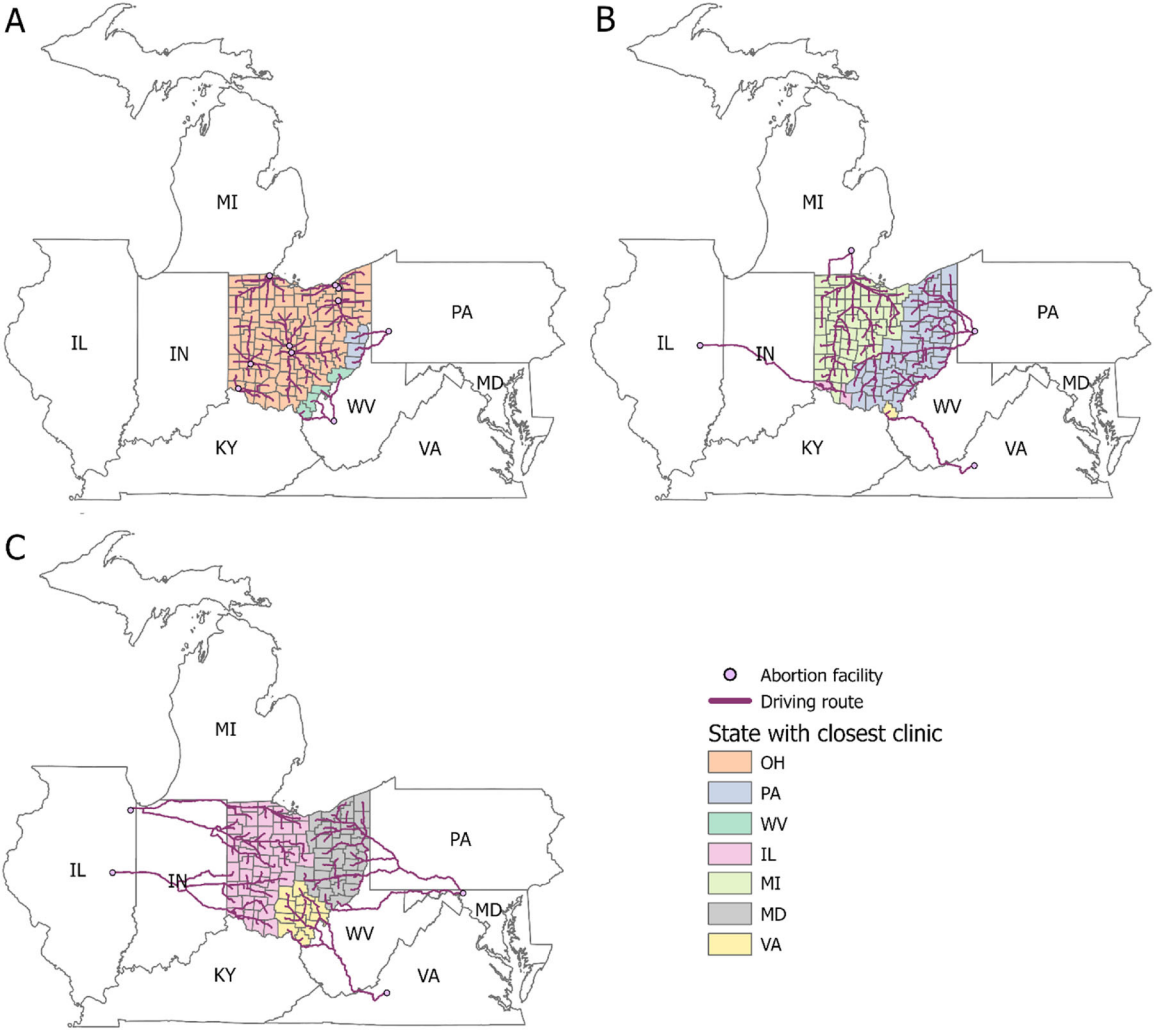
Driving route from county centroid to nearest facility in February 2022 (Panel a), post‐*Roe*, post‐abortion ban if facilities in Michigan and Pennsylvania remain open (Panel b), and post‐*Roe*, post‐abortion ban if facilities in Michigan and Pennsylvania are closed (Panel c), with Ohio counties categorized by the state with the closest facility. OH, Ohio; MI, Michigan; IN, Indiana; IL, Illinois; KY, Kentucky; WV, West Virginia; PA, Pennsylvania; VA, Virginia; MD, Maryland

**FIGURE 2:**
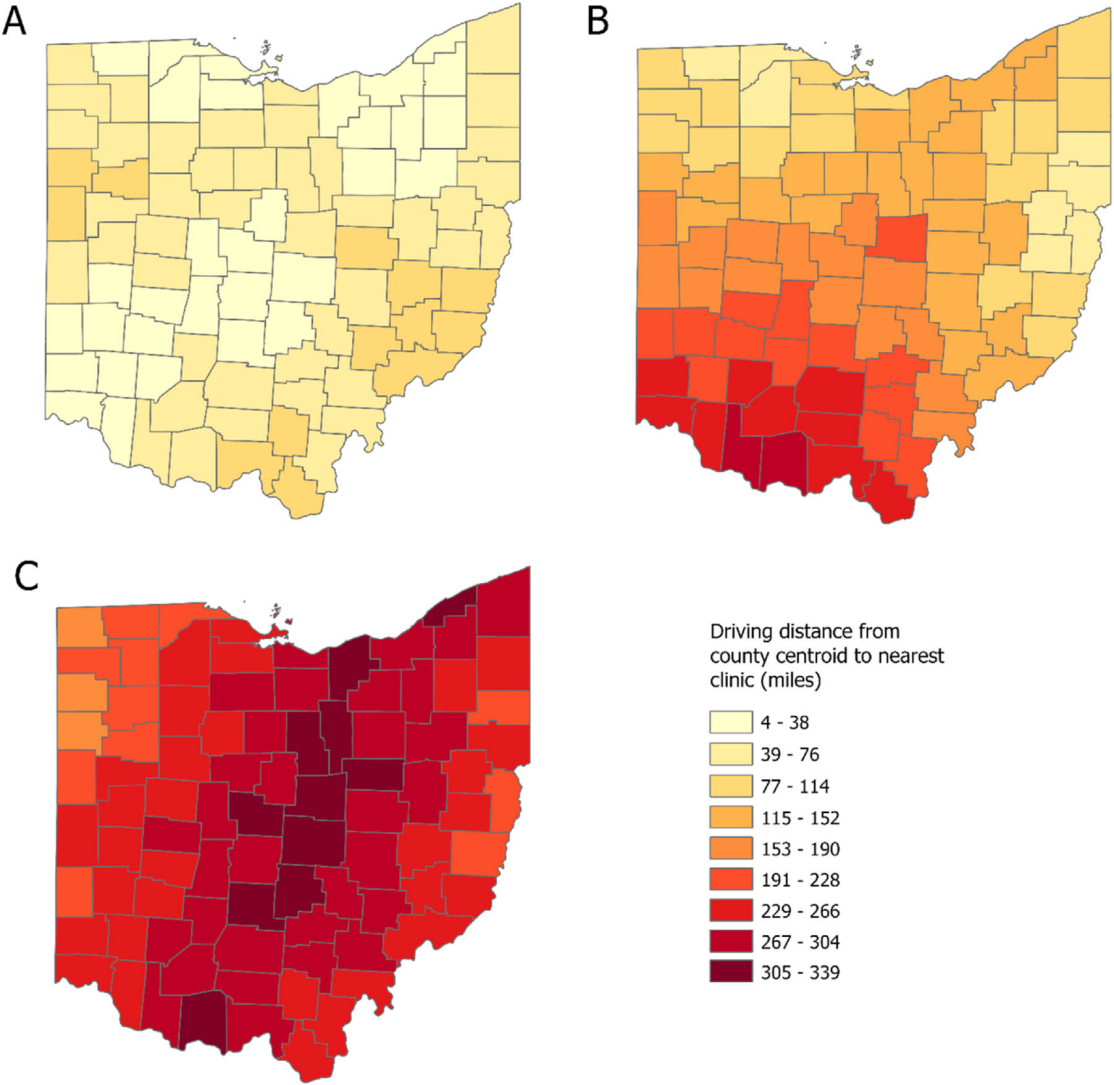
Driving distance from county centroid to nearest facility in February 2022 (Panel a), post‐*Roe*, post‐abortion ban if facilities in Michigan and Pennsylvania remain open (Panel b), and post‐*Roe*, post‐abortion ban if facilities in Michigan and Pennsylvania close (Panel c). Counties are classified by driving distance between county centroid and nearest abortion facility. Panel a represents the distance, as of February 2022, from each county's centroid to the nearest facility, which includes nine Ohio facilities, one Pennsylvania facility, and one West Virginia facility. Panel b represents the distance from each county's centroid to the nearest facility if an Ohio abortion ban takes effect and facilities remain open in Michigan and Pennsylvania; nearest facilities include one Pennsylvania facility, one Illinois facility, one Michigan facility, and one Virginia facility. Panel c represents the distance from each county's centroid to the nearest facility if an Ohio abortion takes effect and facilities in Michigan and Pennsylvania are closed; nearest facilities include two Illinois facilities, one Maryland facility and one Virginia facility. The range of distances in Panel a is 4–99 miles, the range in Panel b is 46–279 miles, and the range in Panel c is 181–339 miles

**FIGURE 3:**
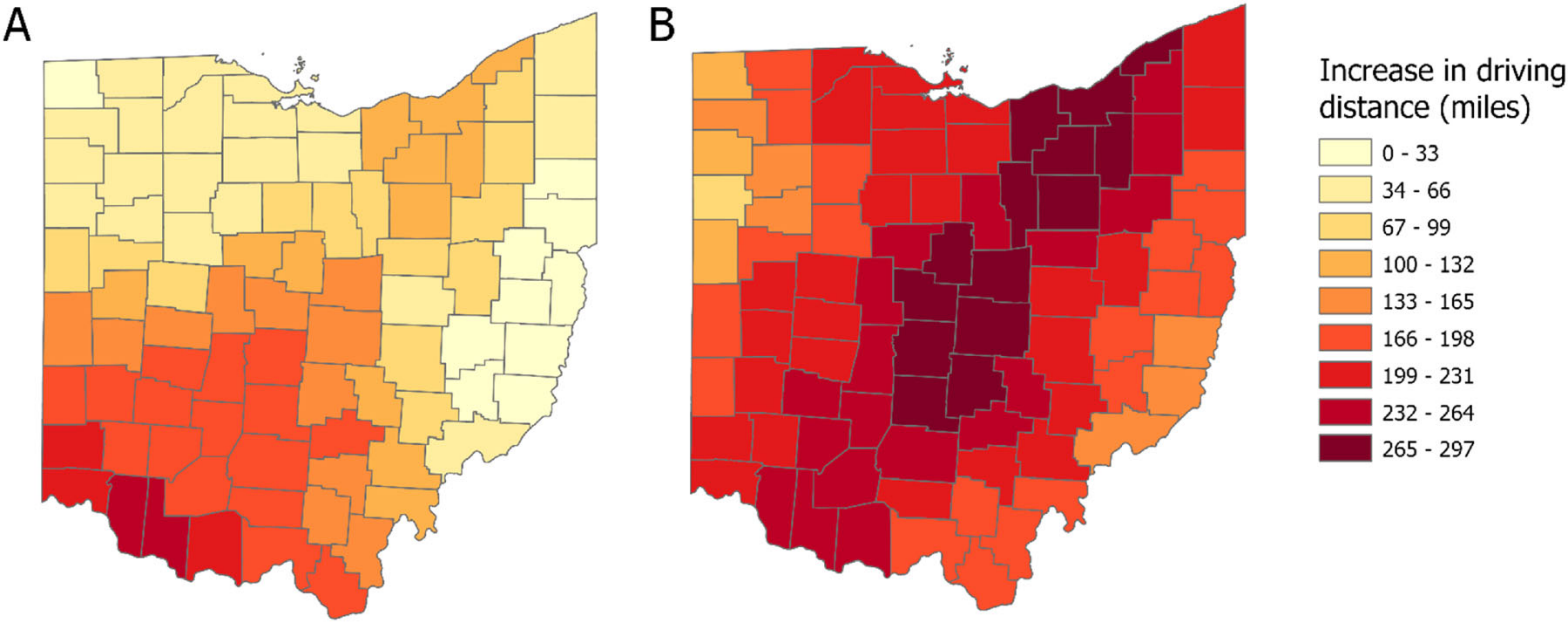
Increase in driving distance from county centroid to closest facility post‐*Roe*, post‐abortion ban if facilities in Michigan and Pennsylvania remain open (Panel a) and if facilities in Michigan and Pennsylvania are closed (Panel b)

Our findings are illuminated via several maps. First, we mapped driving routes from county centroid to the closest facility in all three scenarios, showing how the distribution changes from Scenario A to Scenarios B and C (Figure 1). Second, we grouped counties according to the distance between county centroid and the closest abortion facility for all three scenarios (Figure 2). For ease of visualization, we categorized counties in the maps using driving distance intervals of 38 miles. Because the maximum and/or minimum distances could fall within an interval, we report the maximum and minimum one‐way driving distance for each scenario in the text. Third, we mapped the increase in driving distance from Scenario A to Scenarios B (Figure 3a) and C (Figure 3b).

For all three scenarios, we calculated an average population‐weighted distance for the state of Ohio using the following equation:

$${\mathrm{PWD}}_{\text{Ohio}}=\frac{\sum_{i=1}^{N}P_{i}\times d_{i}}{P_{\text{Ohio}}}$$

where ${\mathrm{PWD}}_{\text{Ohio}}$ is the population‐weighted distance for Ohio, $P_{i}$ is the number of cisgender women of reproductive age (15–44 years old) in county *i*, $P_{\text{Ohio}}$ is the number of cisgender women aged 15–44 years in Ohio, and $d_{i}$ is the driving distance from county centroid to the nearest abortion facility for county *i*. We obtained the number of women aged 15–44 years for each county using 5‐year estimates from the 2019 American Community Survey.^39^ We also calculated population‐weighted driving distances by race (white, Black, Native American, Asian, Pacific Islander, other, multiple races), ethnicity (non‐Hispanic white and Hispanic), and poverty status (living under and above the poverty level) among women of reproductive age in Ohio to see if certain demographic groups are disproportionately impacted. We note that the Census does not report poverty status for all women aged 15–44 years; when calculating percentages that involve poverty status, we used the total number of women aged 15–44 years for whom poverty status was determined, excluding those with missing data only in the population weighted analysis disaggregated by poverty status.

Finally, to understand better dynamics of the effect of these laws, we examined counties with both the greatest changes in driving distance and relatively dense populations of women of reproductive age. Specifically, we highlighted the demographics and changes in driving distance of five counties that: (1) Currently have abortion facilities which would close in a post‐*Roe* scenario; (2) Are geographically distributed across the state; (3) Have relatively sizable populations to be affected by changes in abortion access; and (4) Are more racially diverse compared to Ohio as a whole and thus would help illuminate important differences by sub‐populations that may get washed out when only examining state‐level trends. For these counties, we presented changes in driving distance, as well as demographic breakdowns, of: (1) Reproductive aged women by race, ethnicity, and poverty status; and (2) Number of abortions and abortion rates (number of abortions by 1000 women aged 15–44 years) by race. We note that we examined race as a variable not because of the intrinsic characteristic of race, but rather because of the manifestation of structural inequities that make attention to race relevant. We obtained the number of abortions by county and race from the 2019 Induced Abortion Reports published by the Ohio Department of Health.^40^

## RESULTS

### Statewide impacts

As of February 2022, the nearest abortion facilities to Ohio county centroids include nine facilities in Ohio, one facility in Pennsylvania, and one facility in West Virginia. (Figure 1, Panel a). For most county centroids, the closest facility is in Ohio. In the best‐case post‐*Roe* scenario, where abortion remains available in Michigan and Pennsylvania, abortions would not be accessible in Ohio, Indiana, Kentucky, and West Virginia. Thus, the closest facility for most Ohio county centroids would be in Pennsylvania and Michigan (Figure 1, Panel b). Illinois would host the closest facilities for two county centroids in the southwest part of Ohio, and Virginia would be closest to one county centroid in the southernmost part of Ohio. In the worst‐case post‐*Roe* scenario, where abortion is banned in Michigan and Pennsylvania, the closest facilities to Ohio residents would be in Illinois, Maryland, and Virginia (Figure 1, Panel c).

As of February 2022, all 88 Ohio county centroids have an abortion facility within 99 miles (Figure 2, Panel a). Thirty‐two counties are 4–38 miles from an abortion facility, 43 counties are 36–70 miles away from a facility, and 13 counties are 77–99 miles away from abortion facilities. Centroids for rural counties are currently furthest from abortion facilities. Centroids for counties containing or neighboring urban areas average the shortest driving distances to the nearest facility. The median distance from Ohio county centroid to a facility is 50 miles.

Panels B and C of Figure 2 illustrate the impact to Ohioans were abortion bans to pass and *Roe* to fall. Whereas all Ohioans presently reside in counties with an abortion facility less than 99 miles away, if *Roe* were overturned and an Ohio abortion ban took effect, 62 of Ohio's 88 county centroids would be 115–279 miles away from an open facility in a best‐case post‐*Roe* scenario (Figure 2, Panel b). In this scenario, the median county centroid would be 146 miles from an abortion facility. Counties in northern and eastern Ohio show the least change in distance to nearest facility, because the northern counties neighbor Michigan and the closest facilities to the easternmost counties are already in neighboring Pennsylvania (Figure 3, Panel a). All other counties would have stark increases in distance to the nearest facility. In the worst‐case post‐*Roe* scenario, the centroids for 85 of Ohio's 88 counties would be 191–339 miles away from an open facility, with a median distance of 264 miles (Figure 2, Panel c). In this scenario, residents of all counties would have dramatic increases in distance to the nearest facility, with central counties experiencing the most change (Figure 3, Panel b).

Across all Ohio counties, the current average population‐weighted one‐way driving distance from county centroid to the nearest facility is 26 miles (see Table 1). If *Roe* falls and an Ohio abortion ban is enacted, the average population‐weighted one‐way driving distance from county centroid to the nearest facility would increase about six‐fold, to 157 miles, an increase of about 130 miles in Scenario B. In Scenario C, the average population‐weighted one‐way driving distance would increase more than 10‐fold to 269 miles, a 243‐mile increase. Because of differences in geographic distribution by race, increases in average population‐weighted driving distances are slightly higher among people who are Black (increase of 144 and 255 miles in Scenarios B and C, respectively), Asian (increase of 152 and 260 miles), and Pacific Islander (increase of 145 and 244 miles) as compared to people who are white (increase of 127 miles and 240 miles). Ohioans of color would be disproportionately affected by a state abortion ban.

**TABLE 1:** Population‐weighted driving distance (in miles) for the state of Ohio for women 15–44 years of age overall and by race, ethnicity, and poverty status (*N* = 2,206,822)

	February 2022	Post‐*Roe* (with MI and PA facilities)		Post‐*Roe* (without MI and PA facilities)	
	Population‐weighted driving distance (miles)	Population‐weighted driving distance (miles)	Difference (miles)	Population‐weighted driving distance (miles)	Difference (miles)
Overall (*N* = 2,206,822)	26	157	130	269	243
Race					
White (*n* = 1,727,883)	29	156	127	269	240
Black (*n* = 310,804)	13	157	144	269	255
American Indian (*n* = 4,451)	28	152	124	274	246
Asian (*n* = 68,890)	16	167	152	276	260
Pacific Islander (*n* = 894)	21	167	145	266	244
Other Race (*n* = 26,159)	19	147	128	268	249
Multi‐Racial (*n* = 67,741)	23	155	132	270	247
Ethnicity					
Non‐Hispanic white (*n* = 1,665,286)	30	157	127	269	239
Hispanic (*n* = 99,533)	22	146	123	270	248
Poverty status^a^					
Living below poverty line (*n* = 395,470)	26	154	128	267	241
Living above poverty line (*n* = 1,742,708)	26	157	131	270	243

*Note*: Population weights are at the county‐level and were obtained from 2019 American Community Survey (5‐year estimates).

^a^ The Census does not report poverty status for all women aged 15–44 years.

### County spotlights

Increased travel distance would not be distributed equally across Ohio, nor across Ohioans. We highlight changes in Franklin, Cuyahoga, Hamilton, Montgomery, and Lucas counties, which are highly populated areas (the 1st, 2nd, 3rd, 5th, and 6th most populous counties in the state)^41^ that would experience large changes in driving distance in a post‐*Roe* scenario (Figure 3). These counties contain the cities of Columbus, Cleveland, Cincinnati, Dayton, and Toledo, respectively. Each of these counties currently contains an abortion facility. In Scenario B, people in Franklin County would have an additional 179 miles to drive one way if an abortion ban went into effect, those in Cuyahoga would have 116 additional miles, those in Hamilton would have 222 additional miles, those in Montgomery would have 186 additional miles, and those in Lucas would have 40 additional miles. Differences are generally larger in Scenario C, where those in Franklin County would have 297 additional miles, those in Cuyahoga would have 276 additional miles, those in Hamilton would have 222 additional miles, those in Montgomery would have 225 additional miles, and those in Lucas would have 218 additional miles. In 2019, residents of these five counties accounted for about 60% of the abortions in Ohio: Franklin County 16.7%, Cuyahoga County 23.2%, Hamilton County 11.2%, Montgomery County 6.2%, and Lucas County 3.7%.^40^ In these counties, women of reproductive age are 58%–69% white, 22%–33% Black, and 3%–9% Hispanic (see Table 2); in comparison, Ohio averages 81% white, 12% Black, and 4% Hispanic.^39^ Between 19% and 24% of reproductive aged women in these five counties live under the poverty line (Table 2), compared to Ohio's average of 14%.^39^ The large number of women living below the poverty line in these five counties are likely to be more negatively impacted by abortion ban derived travel burdens due to their financial disadvantage. The abortion rate (abortions per 1000 women of reproductive age) in these five counties is much higher for Black women (between 16.3 and 36.4) and women who are neither Black nor white (between 13.3 and 20.7) than it is for white women (between 4.7 and 7.6) (see Table 3), similar to Ohio as a whole (the abortion rate in Ohio is 4.7 for white women, 26.2 for Black women, and 15.2 for women who are neither Black nor white). Because women of color use abortion care more in these counties, they will be disproportionately impacted in comparison to white women if abortion care is no longer available there.

**TABLE 2:** Women aged 15–44 years overall and by race, ethnicity, and poverty status in Cuyahoga County, Franklin County, Hamilton County, Lucas County, and Montgomery County (*N* = 2,206,822)

	Overall		Cuyahoga county		Franklin county		Hamilton county		Lucas county		Montgomery county	
	*N*	%	*N*	%	*N*	%	*N*	%	*N*	%	*N*	%
Overall	2,206,822	100%	240,880	100%	291,172	100%	164,164	100%	83,830	100%	101,754	100%
Race												
White	1,727,883	78%	138,688	58%	187,303	64%	105,317	64%	57,320	68%	70,468	69%
Black	310,804	14%	78,636	33%	66,320	23%	45,761	28%	18,400	22%	22,990	23%
American Indian	4451	0%	585	0%	691	0%	108	0%	198	0%	260	0%
Asian	68,890	3%	9984	4%	19,295	7%	5957	4%	1901	2%	2905	3%
Pacific Islander	894	0%	100	0%	139	0%	51	0%	16	0%	46	0%
Other race	26,159	1%	4917	2%	5514	2%	1800	1%	2472	3%	1311	1%
Multi‐Racial	67,741	3%	7970	3%	11,910	4%	5170	3%	3523	4%	3774	4%
Ethnicity												
Non‐Hispanic white	1,665,286	75%	128,243	53%	177,810	61%	101,707	62%	53,681	64%	68,208	67%
Hispanic	99,533	5%	17,677	7%	16,314	6%	5659	3%	7157	9%	3666	4%
Poverty status^a^												
Living below poverty line	395,470	18%	49,740	21%	52,612	19%	32,816	20%	19,737	24%	21,031	22%
Living above poverty line	1,742,708	82%	185,882	79%	228,550	81%	127,719	80%	62,301	76%	75,742	78%
Not determined	68,644		5258		10,010		3629		1792		4981	

*Note*: Cuyahoga County is located in northeastern Ohio and contains the city of Cleveland; Franklin County is located in central Ohio and contains the city of Columbus; Hamilton County is located in the southwestern corner of Ohio and contains the city of Cincinnati; Lucas County is located in northwestern Ohio and contains the city of Toledo; and Montgomery County is located in southwestern Ohio and contains the city of Dayton. Data obtained from the 2019 American Community Survey (5‐year estimates).

^a^ The Census does not report poverty status for all women aged 15–44 years.

**TABLE 3:** Number of abortions and abortion rate overall and by race in Cuyahoga County, Franklin County, Hamilton County, Lucas County, and Montgomery County (*N* = 18,913)

	Overall		Cuyahoga County		Franklin County		Hamilton County		Lucas County		Montgomery County	
	*N*	Rate^a^	*N*	Rate^a^	*N*	Rate^a^	*N*	Rate^a^	*N*	Rate^a^	*N*	Rate^a^
Overall	18,913	8.6	4381	18.2	3166	10.9	2124	12.9	695	8.3	1176	11.6
Race												
White	8198	4.7	1052	7.6	1088	5.8	626	5.9	272	4.7	422	6.0
Black	8152	26.2	2863	36.4	1484	22.4	1227	26.8	299	16.3	644	28.0
Other race	2563	15.2	466	19.8	594	15.8	271	20.7	124	15.3	110	13.3

*Note*: Cuyahoga County is located in northeastern Ohio and contains the city of Cleveland; Franklin County is located in central Ohio and contains the city of Columbus; Hamilton County is located in the southwestern corner of Ohio and contains the city of Cincinnati; Lucas County is located in northwestern Ohio and contains the city of Toledo; and Montgomery County is located in southwestern Ohio and contains the city of Dayton. Data obtained from the 2019 Induced Abortion Report published by the Ohio Department of Health.

^a^ Abortion rate is calculated as the number of abortions per 1000 women aged 15–44 years.

## DISCUSSION

If an abortion ban in Ohio were to go into effect, Ohioans will have to go out‐of‐state for facility‐based abortion care, resulting in huge increases in travel distance. The median one‐way driving distance from county centroid to facility would rise from 50 to 146 miles, or 264 miles depending on accessibility in Michigan and Pennsylvania, in a post‐*Roe* scenario. With population‐weighted averages, we evaluated the combination of *distance* and the *number of people* who would be impacted, and we found that the impact of pre‐ to post‐*Roe* change is even greater when the geographic distribution of women of reproductive age is taken into account. The post‐*Roe* population‐weighted one‐way driving distance would increase from 26 to 157 miles (best case) or 269 miles (worst case), 6‐ and 10‐fold increases, respectively. We see slightly increased travel distances for people of color as compared to white people and note that even if driving distances increased uniformly across racial groups, the burdens of those increases would not be equally experienced. For example, for individuals facing financial insecurity, those with jobs from which they cannot take time off, or those who cannot find childcare, an increase in driving distance to receive abortion care would be more difficult and costly.

Currently, all of Ohio's country centroids are within 99 miles of a facility. In a post‐*Roe* scenario in which SB 123/HB 598 and/or HB 480 are in effect, driving distance would increase for people in 84 of the state's 88 counties in a best‐case scenario; driving distance would increase even more dramatically if Michigan and Pennsylvania were also to ban abortion. In particular, residents of mid‐ and southwestern Ohio counties, who make up almost half of Ohio's reproductive‐aged women,^39^ would be critically impacted by these changes given their location relative to abortion facilities now and in a post‐*Roe* scenario. In the best‐case scenario, Ohioans in mid‐ and southwestern counties would live 153–279 miles from the nearest abortion facility. If Michigan and Pennsylvania lose access to abortion, all Ohioans would have to travel 181–339 miles to access abortion care, with central counties experiencing the greatest travel burden (267–339 miles). Our analysis of Franklin, Cuyahoga, Lucas, Hamilton, and Montgomery counties emphasizes that Black women and women who identified as neither white nor Black are more likely to have abortions and thus would be more disadvantaged, as a group, by changes in these counties. Similarly, given that nationally almost half of abortion patients were estimated to have family incomes that were less than 100% the poverty level in 2014,^42^ expanded barriers to care via increased travel would fall on those who already face numerous other barriers.

Increased driving distance translates into increased cost as well as increased time. Given the 2022 federal travel reimbursement rate of $0.585/mile (which includes costs of fuel, insurance, and wear‐and‐tear on automobile),^43^ driving the current longest one‐way trip to a facility costs approximately $58. In the post‐*Roe*, post‐abortion ban scenario, this cost would increase to up to $163 in the best case‐scenario and up to $198 in the worst‐case scenario per one‐way trip, a substantial burden for low‐income Ohioans who are least able to afford the cost. Travel by car also may necessitate paying for tolls, parking, and an overnight stay where the abortion will take place, increasing costs associated with seeking abortions.

In addition to more costs, delays, and reduced care options, increased barriers to abortion access will inevitably result in some people to not be able to obtain their desired abortion, as increased distance to abortion care creates barriers for people seeking care^5^, ^13^, ^14^, ^15^, ^16^ and is associated with lower rates of utilization.^44^ These barriers disproportionally affect people from lower socioeconomic backgrounds and people of color.^10^, ^18^, ^19^ Denial of a wanted abortion can cause severe negative consequences for the pregnant person, such as increase in short‐term anxiety, stress, and low self‐esteem^25^; poorer physical health^24^, ^26^; economic insecurity^27^, ^28^; and greater exposure to violence from an intimate partner.^29^

Actual travel distances could be greater than our estimates for many people—particularly for residents of rural counties, who may have to travel even farther to reach the nearest highway—because our analysis computed driving distances for each county starting in the county centroid. We note several additional burdens not captured in our analysis of increases in distance to travel. First, many patients do not have access to their own vehicles; relying on public transportation for longer distances would take far more time and be more logistically complicated. Second, abortion facilities do not all have the same gestational limits nor provide the same services,^45^ so distance traveled by people who have specific parameters for care may be far greater. Third, we include facilities that do not provide instrumentation abortions in these analyses, which may underestimate how far people in need of this method, or receiving care at later gestations, must travel. Fourth, we cannot account for the fact that people might also choose to travel further than the first available facility for abortion care.^46^ Finally, in a post‐*Roe* scenario, abortion facilities in states where abortion will remain accessible will likely experience an influx of patients traveling from states where abortion is banned, leading to facility congestion.^47^ Thus, open abortion facilities may not be able to handle the increase in patient demand and wait times may be longer than usual.^48^, ^49^ The resulting expanded wait times may preclude some people from obtaining care within the gestational limit of the facility.

An abortion ban would substantially increase driving distance to obtain abortion services for most Ohioans, forcing them to travel across state lines to access abortion care. Many Ohioans, especially those living in rural areas, already experience the substantial consequences of long travel distances for abortion care.^10^, ^38^ Our projections suggest that SB 123/HB 598 and/or HB 480 would force all Ohioans into this reality, one which compounds the burden for those who already experience the most inequities in access to healthcare.

## CONFLICT OF INTEREST

The authors declare no conflict of interest.
